# Diplomacy and Health: The End of the Utilitarian Era

**DOI:** 10.15171/ijhpm.2016.155

**Published:** 2017-01-08

**Authors:** Sebastian Kevany, Marcus Matthews

**Affiliations:** ^1^University of California, San Francisco, CA, USA.; ^2^Amur Consultancy, Dublin, Ireland.

**Keywords:** Diplomacy, Cost-Effectiveness, Threshold, Resource Allocation

## Abstract

Cost-effectiveness analysis (CEA), as a system of allocative efficiency for global health programs, is an influential criterion for resource allocation in the context of diplomacy and inherent foreign policy decisions therein. This is because such programs have diplomatic benefits and costs that can be uploaded from the recipient and affect the broader foreign policy interests of the donor and the diplomacy landscape between both parties. These diplomatic implications are vital to the long-term success of both the immediate program and any subsequent programs; hence it is important to articulate them alongside program performance, in terms of how well their interrelated interventions were perceived by the communities served. Consequently, the exclusive focus of cost-effectiveness on medical outcomes ignores (1) the potential non-health benefits of less cost-effective interventions and (2) the potential of these collateral gains to form compelling cases across the interdisciplinary spectrum to increase the overall resource envelope for global health. The assessment utilizes the Kevany Riposte’s "K-Scores" methodology, which has been previously applied as a replicable evaluation tool^1^ and assesses the trade-offs of highly cost-effective but potentially "undiplomatic" global health interventions. Ultimately, we apply this approach to selected HIV/AIDS interventions to determine their wider benefits and demonstrate the value alternative evaluation and decision-making methodologies. Interventions with high "K-Scores" should be seriously considered for resource allocation independent of their cost-effectiveness. "Oregon Plan" thresholds^2^ are neither appropriate nor enforceable in this regard while "K-Score" results provide contextual information to policy-makers who may have, to date, considered only cost-effectiveness data. While CEA is a valuable tool for resource allocation, its use as a utilitarian focus should be approached with caution. Policy-makers and global health program managers should take into account a wide range of outcomes before agreeing upon selection and implementation.

## Background

### A Challenge to Cost-Effectiveness Analysis

In recent decades, cost-effectiveness analysis (CEA) has become increasingly important in both technical and allocative resource allocation decisions for global health interventions.^3^ This includes, but is not limited to, the determination of which programs to support, in which places and focusing on which populations, based on cost per unit of currency involved against outcomes such as HIV/AIDS infections averted, quality-adjusted life years gained, or disability-adjusted life years lost.^4^ Such utilitarian approaches, while valuable in the technical efficiency realm, have been considered short-sighted and narrow in “real–world” scenarios, especially those in which cultural, social, religious, diplomatic, equity, accessibility, and political considerations have to be taken into account.^5^ How does CEA decision-maker respond, for example, to situations in which tides of popular support for less cost-effective interventions, such as antiretroviral therapy for HIV/AIDS, result in an increase in funding for global health,^6^ and thereby potentially saving more lives than if optimal utilitarian interventions were exclusively used?

### Global Health Intervention Value

Assessing the relative value or worth of global health interventions begins with the development of hypothetical comparisons. A range of highly cost-effective interventions for HIV/AIDS are, of course, already in existence.^7^ To date, few of these have been formally assessed from a foreign policy perspective.^8^ However, certain features of contemporary interventions suggest possible foreign policy advantages or threats.^1^ In the case of HIV/AIDS, behavioral or surgical interventions are frequently in conflict with local traditions and societal norms; from the social, religious or cultural viewpoints.^9^ While such interventions may be highly cost-effective, how much attention from their advocates has gone in to the challenges to local health traditions — notwithstanding possible downstream health effects — in the developing world’s primary healthcare context? Similarly, the promotion of other HIV/AIDS interventions has, to date, paid little heed to the challenges that this brings about on the non-health level.^10^

### Diplomatic Versus Economic Value

To assess the total utility of global health interventions and capture the wider interrelated community health and non-health benefits, which is of real interest to program donors; it is necessary to consider a quantitative model that is designed to capture the broader socio-economic implications. The “Kevany Riposte” is a recently-developed and published CEA tool that assigns numerical values to the diplomatic worth (or threats) of global health interventions.^1^ The Kevany Riposte incorporates the following 10 assessment criteria when a particular intervention’s inherent design is being analyzed (Table 1).

**Table 1:** Criteria and Non-exhaustive Outline of Themes Assessed and Questions Evaluated

**Criterion**	**Description of Themes (Not Exhaustive)**
Neutrality	How tailored is the intervention to the recipient’s society, religious practices, cultural values?
Visibility	How visible are the source funding organizations?
Sustainability	Can the intervention be financially supported by the recipient after the funding period? Can the intervention be transferred?
Effectiveness	Has the intervention and its results been scientifically validated? Are there measures in place to deal with constrained budgets?
Adaptability	Can the intervention respond to unforeseen health needs? Does the intervention have positive externalities? Have communities had an input?
Accountability	Does the intervention produce regular results from communities that are verifiable? Is an M&E philosophy prevalent and is corruption combatted?
Partnerships	Does the intervention promote institutional partnerships: national and regional? Do intervention staff receive guidance on international standards?
Economic, Political, Environmental and Social Effects	Does the intervention contribute to wider economic growth? Does the intervention promote political stability? Does the intervention increase dignity and self-worth amongst recipients? Does the intervention utilize public space appropriately? Does the intervention damage the environment?
Interdependence	Is the intervention coordinated with the aims of other programs? Does the intervention complement or operate in tandem with other interventions?
Training	Have intervention staff been trained? Is the training qualification recognized? Have staff received training to deal with cultural and religious customs?

Abbreviation: M&E, Metaphysics and Epistemology.

In each instance, the specific criterion is graded on a mathematical benefit-threat spectrum, which results in a “K-Score” that quantifies the potential risks and rewards of the criteria that constitute a particular intervention. When the K-Scores are summated, they are contextualized against the “Kevany Threshold” (KT), which is an effectiveness quorum that can be optimized depending upon the donor’s effectiveness tolerance. The K-Score’s inherent threat-benefit calculations are, therefore, mathematically rated accordingly (Table 2).

**Table 2:** Scoring and Results for K-Score Classifications

** Classification **	** Explanation**	**Score**
Highly advantageous	Intervention program displays clear and significant value from the diplomatic or foreign policy perspective.	+2
Moderately advantageous	Intervention program displays some strengths in advancing diplomatic or foreign policy goals.	+1
Acceptable, neutral, or not relevant	Intervention attains diplomatic or foreign policy minimum standards.	0
Not applicable	Intervention program does not operate in the context of this classification (or sub-classification).	0
Potential moderate threat	Intervention program may constitute a threat to diplomatic or foreign policy goals.	−1
Potential significant threat	Intervention program constitutes a clear and significant threat from the diplomatic or foreign policy perspective.	-2

The result from the application of the Kevany Riposte is an overall clearer understanding of an intervention’s cost-effectiveness and potential externalities. Thus, the Kevany Riposte and the resulting positioning with respect to the KT can offer a much better understanding of a particular intervention’s diplomatic value to a donor’s philosophy, national values, and overall foreign policy agenda.

### Balancing Diplomatic and Economic Value With the Kevany Riposte

The assessment of global health interventions that fail to achieve the KT results in a hypothetical model that (1) forces an intervention’s rejection from the resource allocation pantheon or (2) necessitates the intervention’s modification to adhere to the diplomatic criteria listed above. In practice, this would involve the application of a KT to a selection, or the entire range, of HIV/AIDS interventions currently in use. Such a process would take place both via desk reviews and at the field level, focusing on those classifications and sub-classifications under which the intervention registered potential, moderate or severe diplomatic threats. For example, intervention timelines and the feasibility of long-term handover to local actors would be guaranteed, in advance of intervention implementation, to be within the capacity of local actors in the long-term.^11^ Similarly, there is an increasing consensus that global heath interventions which stand to challenge local healthcare as well as other traditions or practices should be designed and delivered on a highly collaborative and interactive basis with recipient countries, local communities and other stakeholders.

The result of such diplomatic “screening” procedures for global health programs will be to ensure that, on a *prima facie* basis, all interventions are both cost-effective and diplomatically-effective – ideally, without sacrificing either health or non-health gains, but nonetheless providing analytical tools to respond to ethical dilemmas related to situations in which such trade-offs have to occur. The combination of such considerations stands to improve program implementation, uptake, utilization, donor support, and recipient recognition and appreciation of donor efforts.^12^ Let us consider a concrete worked example showing a plausible (not clearly unethical) cost-effective intervention that can be rejected (or severely modified) and swapped out based on diplomatic criteria.

## Methods

### Comparing Diplomatically and Economically-Effective Choices

Our example focuses on, firstly, a global health intervention for HIV/AIDS that is effective, efficacious and cost-effective; in both the controlled (trial) and applied (field) contexts (“Intervention A”), and that is implemented based on surgical procedures (eg, voluntary adult male circumcision). The context is a low-income, developing country setting with limited educational opportunities and high levels of religious practices and awareness, as well as a significant focus on traditional (even tribal) values related to sexual health and gender norms. These norms are not considered damaging from western perspectives; rather, they are neutral in terms of social progressiveness, recognizing the multifarious global health interventions that address, in concert with each other, both health and (for example) oppressive gender practices that are to be welcomed and supported. Our cost-effectiveness ratio for this intervention is approximately US$3000 per HIV infection averted, both with a “K-Score” of 5 out of a possible 10, with 3 criteria classified as “significant diplomatic threats”: namely, sustainability, adaptability, and neutrality.

Our comparator intervention (“Intervention B”) is implemented via a combination of voluntary counseling and testing, post-test support services, antiretroviral treatment, and community mobilization, as well as utilizing strategies such as; “abstinence, be faithful, and condomize” (ABC), which represent interventions with both health and non-health agendas,^13^ and scores highly on the K-Score (9 out of 10, with 3 criteria classified as “significant diplomatic advantages”) but is less cost-effective (US$5000 per HIV infection averted). Such an intervention has been initially evaluated from the outset of study design for effect on diplomatic and foreign policy outcomes, as well as standard health and medical metrics.^1^ More specifically, challenges to (non-damaging) local customs have been mitigated, sustainability has been ensured; downstream and side-effects have all been considered as part of the original assessment plan. Effects on local perceptions of donors, cultural, and religious acceptability, and the anticipation of long-terms intervention issues have also been considered on a *prima facie* basis.

## Results

Finally, we take the United Nations (UN) international statistical standard for population health metrics of 100 000 susceptible persons suffering from a generalized HIV epidemic (prevalence 10% and incidence 2%). Total number of HIV infections, therefore, currently stand at 10 000 and increase (excluding annual HIV-related deaths) at a rate of 2000 per year. The implementation of intervention A, under a fixed budget of US$1 000 000, would, in this theoretical framework, avert 333 HIV infections in the first year of implementation or 16.6% of new infections. Conversely, intervention B^ [1]^ would avert 200 infections or 10%. Our question then becomes: is the difference in the number of HIV infections averted (133 infections or 6.6% of new infections) offset by diplomatic gains, including but not limited to; (1) their potential to attract additional funds to intervention B, thereby increasing the funding envelope (eg, increases in support for antiretroviral and other global health interventions at the turn of the century),^14^ (2) the possible health gains consequent on increased long-term utilization via the performance of the sustainability, visibility, and cultural acceptability criteria^15^ for intervention B, and (3) at the most abstract level, the (nebulous and possibly unquantifiable) health gains and lives saved as a result of improved international relations between donor and recipient countries.^16^

If these possible benefits can be shown to equal or exceed the 133 HIV infections that the original choice of intervention B failed to avert, the case for preferring investment in intervention B is strengthened. Let us assume that, for example, the diplomatic success of the intervention resulted in a doubling of funding for intervention B (from US$1 000 000 to US$2 000 000), thereby also doubling the number of HIV infections averted (from 200 to 400). This means that, in comparison to the choice of staying with the “less diplomatic” intervention A, an additional 67 HIV/AIDS infections (or 20% of all new infections) are averted in the first year of implementation. Such increases in funding and support for interventions that capture both donor and recipient imaginations, as well as “hearts and minds,” is not unprecedented (witness the dramatic mobilization of funding for antiretroviral treatment during the 1990s and early 2000s, for example^17^).

## Discussion

### K-Scores and Cost-Effectiveness Ratios

An additional assumption is the effect of improvements in K-Scores on cost-effectiveness ratios. Improved K-Scores are not connected to the cost of the intervention (either absolute cost or cost per unit of output or outcome). Rather, these scores are linked to outcomes. For example, if we accept an increase in funding for the intervention by a factor of 20% — as a possibly conservative estimate in the context of the quadrupling of funding for global health interventions in recent years — for each additional K-Score point, outcomes or outputs improve in a directly corresponding manner. In this example, a differential in K-Scores of 4 implies an increase in funding for intervention B of 80%. Based on our initial budget of $1 000 000 for intervention B, this would, therefore, increase to $1 800 000. This improved budget would, therefore, generate a total of 360 HIV infections averted.

### Advantages and Disadvantages

As an alternative or complement to traditional CEA for global health, the development and application of diplomatic thresholds has both advantages and disadvantages. Positives include the development and selection of more sensitive, sustainable and “diplomatic” global health interventions that advance, for example, international relations, cooperation stability, security and conflict resolution – the broader interests of the global community – without sacrificing the primary altruistic, humanitarian, and development goals of international medical assistance measures. Possible disadvantages include the need for additional design considerations for global health interventions that integrate these concerns, rather than, as in the past, simply developing an effective or cost-effective intervention and proceeding directly to implementation. The feasibility of these pre-implementation checks should also be considered – is it realistic for bilateral and multilateral donors, as well as non-governmental organizations (NGOs), to undertake “diplomatic evaluation” checks, via a checklist or electronic app, on their efforts on both a *pre-hoc* and *post-hoc* basis? Many would, however, consider these added bureaucratic and administrative hurdles a small price to pay for the dual advancement of diplomatic and development goals in a synergistic fashion.

## Conclusions

In this example, the KT is based on an increase in funding envelopes contingent on the fulfillment of diplomatic criteria. It is hard, but not impossible, to support this assertion. It is also, perhaps, no coincidence that the ascendancy of global health interventions which are likely to score highly in terms of K-Scores (eg, antiretroviral treatment and voluntary counseling and testing) coincided with a period of dramatic growth in global health funding.^18^ Conversely, in latter years, the focus towards less emotive efforts was linked, again perhaps coincidentally, with more cost-effective but potentially less diplomatic efforts.^19,20^ The “threshold” is, therefore, based on considerations such as the capacity to attract funding. In practice, the KT can, therefore, be defined as “the point at which diplomatic outcomes, including sustainability, cultural awareness and other considerations, increase intervention funding, momentum, utilization and support to such an extent that the total number of HIV infections averted equals or exceeds those of alternative HIV infections, with better cost-effectiveness in controlled, rather than ‘real world,’ long-term settings.” The consideration of the KT in resource allocation decisions and global health intervention selection, therefore, stands to simultaneously advance diplomatic and health goals, at the expense of neither.

## Ethical issues

Not applicable.

## Competing interests

The authors declare that they have no competing interests.

## Authors’ contributions

Conception and design, acquisition of data, analysis and interpretation of data, drafting of the manuscript, statistical analysis: SK and MM. Critical revision of the manuscript for important intellectual content; Administrative, technical, or material support: MM. Supervision: SK.

## Authors’ affiliations

^1^University of California, San Francisco, CA, USA. ^2^Amur Consultancy, Dublin, Ireland.

## Endnote


[1] The “lifetime cost savings” for Interventions A and B are assumed, for the purposes of this model, to be the same.

